# Successive nitric oxide and lipoic acid priming mitigates [ZnO]NPs toxicity in wheat: experimental and DFT insights

**DOI:** 10.1038/s41598-026-56678-0

**Published:** 2026-06-18

**Authors:** R. M. El Shazoly, A. M. El Zohary, Muhammad M. El-Sayed, A. A. Othman, A. A. K. Mohammed, R. Abdel-Basset

**Affiliations:** 1 Botany and Microbiology Department, Faculty of Science, New Valley University, Al-Kharja, New Valley, 72511 Egypt; 2 Technology, Engineering and Mathematics School, Assiut, 71511 Egypt; 3 Physics Department, Faculty of Science, Assiut University, Assiut, 71515 Egypt; 4 Chemistry Department, Faculty of Science, Assiut University, Assiut, 71516 Egypt; 5 Botany and Microbiology Department, Faculty of Science, Assiut University, Assiut, 71516 Egypt

**Keywords:** Seed treatment, Successive priming, Redox signaling, Nanotoxicity, DFT, PBE0-D3BJ, Radical scavenging, Sustainable agriculture, Biochemistry, Biotechnology, Plant sciences

## Abstract

The global food security is threatened by the accumulation of anthropogenic nanomaterials in the environment. We investigated the phytotoxic effects of elevated zinc oxide nanoparticles ([ZnO]NPs: 300–400 mg L⁻^1^) on wheat (*Triticum aestivum* L.) and evaluated a novel detoxification strategy using successive redox-priming. Wheat caryopses were primed with alpha-lipoic acid (LA) and sodium nitroprusside (SNP, a nitric oxide donor) either individually or in successive sequences (LA→SNP and SNP→LA). Exposure to [ZnO]NPs resulted in significant growth inhibition, reduction of biomass and degradation of chlorophyll. However, successive priming, particularly the LA+SNP combination, significantly attenuated these negative effects. This treatment effectively restored seedling growth by creating a strong antioxidant shield, greatly increasing the activities of SOD, POD, CAT, and APX, and raising metal chelating activity to 98.1%. Furthermore, Density Functional Theory (DFT) calculations, including HOMO–LUMO and molecular electrostatic potential (MEP) analyses, gave mechanistic insights into the molecular interactions of the priming agents and ZnO, highlighting their role as potent radical scavengers and stabilizers. The results indicate that a successive redox priming is an effective and sustainable approach to enhance crop tolerance to high concentrations of nanoparticle toxicity.

## Introduction

Zinc (Zn) is an essential micronutrient for higher plants, serving as a structural and functional cofactor for numerous proteins and enzymes. It is essential for preserving biomembrane integrity, modulating gene expression, and maintaining photosynthetic efficiency^1,2^. In the age of nanotechnology, zinc oxide nanoparticles ([ZnO]NPs) have emerged as versatile engineered nanomaterials (ENMs) that can be used in many different fields, such as electronics, industrial catalysis, and biotechnology^3,4^. While exhibiting potential as ‘nano-fertilizers’ at low levels, their growing production —with an estimated 0.3 million tons entering soil and aquatic systems annually—poses an escalating environmental threat^5–8^. The accumulation and subsequent leakage of these nanoparticles into the rhizosphere pose severe risks to plant health and, consequently, the entire food chain, making human health a final recipient of such hazards^9^. To lessen the harmful effects of nano-phytotoxicity, seed priming has been identified as a long-lasting and effective way to boost seedling health and stress resistance^10–12^. This method starts metabolic processes that happen before germination, like fixing DNA and making new antioxidant enzymes, which help plants deal with stressors that come later more effectively^13–15^. Recent progress in redox priming has unveiled molecules such as sodium nitroprusside (SNP) and α-lipoic acid (LA) as formidable elicitors. Nitric oxide (NO), synthesized from SNP, is a gaseous signaling molecule that regulates extensive physiological pathways, including stomatal control and reactive oxygen species (ROS) scavenging^16–18^. Conversely, LA is a distinctive pleiotropic antioxidant that serves as a redox buffer and a precursor for hydrogen sulfide (H_2_S) signaling^19–22^. The interaction between NO and H_2_S is postulated to regulate cellular homeostasis through post-translational changes of cysteine-based proteins; however, the synergistic mechanisms underlying their protective effect against high-dose nano-stress remain poorly understood^23–29^.

Current literature predominantly emphasizes the stimulatory effects of low-dose [ZnO]NPs however, there exists a significant knowledge deficit concerning the detoxification mechanisms when these particles surpass a toxic threshold. Our prior research has illustrated the dual characteristics of ZnO, shifting from a biofortificant at lower doses^30^, to a prospective toxin at elevated concentrations. Additionally, we have recently demonstrated that successive priming—the sequential application of various priming agents—yields a more effective protective effect than individual treatments by inducing a ‘stress memory’ in the seeds^31^.

While individual priming with SNP or LA has been studied, the potential synergistic effects of their successive application remain largely unexplored in the context of [ZnO]NPs toxicity. This study aims to bridge this gap by assessing the effect of priming order (LA→SNP vs. SNP→LA) on the internal redox environment of wheat grains. We hypothesize that successive redox-priming will activate a stronger antioxidant defense system than single treatments by a hierarchical signaling cross-talk improving radical scavenging and membrane stability. Furthermore, we expect that DFT calculations will provide a quantum-mechanical explanation for these interactions, revealing the specific molecular affinities that drive the detoxification process.

## Material and methods

### Source and characterization of [ZnO]NPs

Commercial ZnO (Chem. lab nv, Belgium 99.5% purity) was processed via ball milling to obtain [ZnO]NPs with a mean particle size of 37±2.6 nm. A stock suspension (400 mg L⁻^1^) was prepared in deionized water and sonicated for 30 min (40 kHz) to minimize agglomeration and ensure homogenous dispersion before diluting to the required test concentrations^32^.

### Plant material and priming protocol

Wheat (*Triticum aestivum* L., cv. Giza 168) caryopses were surface sterilized in 10% H₂O₂ for 20 min. The concentrations (300 and 400 mg L⁻^1^) were selected based on preliminary visual screening that showed significant growth retardation (moderate to severe phytotoxicity) without reaching total lethality, and intended to mimic severe environmental contamination situations or ‘hotspots’ in industrial and agricultural runoff locations. However, even if lower concentrations are often reported in the literature, the testing of these critical thresholds allows a robust evaluation of the protective capacity of successive priming under severe phytotoxic stress, allowing for the investigation of acquired tolerance mechanisms.

The caryopses were categorized into different treatment groups. For single priming, grains were immersed in either 2 mM α-lipoic acid (LA) or 0.5 mM Sodium Nitroprusside (SNP) for 12 h in complete darkness at 25±2 °C. For successive priming (LA+SNP or SNP+LA), the grains were sequentially treated with the first agent for 12 h, briefly air-dried on filter paper, followed by a 12-h immersion in the second agent in the dark. This sequential approach aimed to evaluate the synergistic potential of sulfur and nitric oxide signaling.

### Sand matrix and growth conditions

Grains were sown in quartz sand (pre-treated with 1% HCl and rinsed) at a density of 15 grains per pot (700 g sand). Pots were maintained at 100% field capacity (~11% water content). After germination, seedlings were thinned to 10 per pot.

### Harvest and growth measurement

After one month, at the experiment’s end, harvested shoots and roots were weighed to determine fresh weight (FWt), then oven-dried at 70 °C for 48 h to obtain a constant dry weight (DWt) and calculate water content. Plant growth parameters, including shoot height and root length, were also recorded. Freshly harvested shoots and root fractions were stored immediately at -80ºC for further analysis.

### Photosynthetic pigments

Extracted from fresh leaves using 95% (v/v) ethyl alcohol at 60°C. Absorbance was recorded at 663, 644, and 452 nm, and concentrations were calculated according to Lichtenthaler (1987)^33^.

### Enzymatic antioxidants

Fresh tissues (0.5 g) were homogenized in 50 mM Tris-HCl buffer (pH 7.0) containing 1 mM EDTA and 3 mM MgCl₂^34^. Superoxide dismutase (SOD) activity was determined by measuring the autoxidation of epinephrine^35^. Activity was expressed as µM adenochrome min^-1^mg^-1^ protein (UE mg^-1^ protein). Catalase (CAT) activity was determined by monitoring the decrease in H_2_O_2_ absorbance at 240 nm^36^. The activity of (POD) activity was assayed by following the increase in absorbance at 470 nm^37^. The activity of (APX) was conducted by recording the decrease in reading due to ascorbic acid oxidation in 290 nm for 3 min^38^.

### Nonenzymatic antioxidants

Phenolics were determined using the Folin-Ciocalteu’s phenol reagent^39^. Total Phenolic content was expressed as µg gallic acid equivalent (GAE) g^-1^ FWt. The total content of flavonoids was assayed as described by Blois (1958)^40^. Quercetin was used to prepare the standard curve and total flavonoids were calculated as µg g^-1^ FW. 2,2-diphenyl-1-picrylhydrazyl (DPPH) stable free radical scavenging activity was determined^41^. Ascorbic acid and BHT were used as the reference materials. The radical scavenging activity (I %) was calculated using the following equation: Reducing power was assayed^42^. The results were expressed as mg g^-1^ FW using ascorbic acid to prepare a standard curve.

$$I \left( \% \right)=( {\mathrm{A}\mathrm{b}\mathrm{s}}_{\mathrm{C}\mathrm{o}\mathrm{n}\mathrm{t}\mathrm{r}\mathrm{o}\mathrm{l}}-{\mathrm{A}\mathrm{b}\mathrm{s}}_{\mathrm{S}\mathrm{a}\mathrm{m}\mathrm{p}\mathrm{l}\mathrm{e}} )/{\mathrm{A}\mathrm{b}\mathrm{s}}_{\mathrm{C}\mathrm{o}\mathrm{n}\mathrm{t}\mathrm{r}\mathrm{o}\mathrm{l}} \mathrm{x} 100$$

### Free radicals scavenging abilities

Hydroxyl radical scavenging percent was assayed^43^. The absorbance of extracts was measured at A532 nm against a blank containing deoxyribose and buffer. The inhibition percentage (I %) was calculated as radical scavenging activity as follows:

$$I \left( \% \right)=( {\mathrm{A}\mathrm{b}\mathrm{s}}_{\mathrm{C}\mathrm{o}\mathrm{n}\mathrm{t}\mathrm{r}\mathrm{o}\mathrm{l}}-{\mathrm{A}\mathrm{b}\mathrm{s}}_{\mathrm{S}\mathrm{a}\mathrm{m}\mathrm{p}\mathrm{l}\mathrm{e}} )/{\mathrm{A}\mathrm{b}\mathrm{s}}_{\mathrm{C}\mathrm{o}\mathrm{n}\mathrm{t}\mathrm{r}\mathrm{o}\mathrm{l}} \mathrm{x} 100$$

Radical scavenging percent for H_2_O_2_ was assayed^44^. Sodium pyruvate was used as the reference compound. The absorbance of extracts was measured at A560 nm of complex ferric-xylenol orange. The inhibition percentage (I %) was calculated as radical scavenging activity as follows:

$$I \left( \% \right) = ({\mathrm{A}\mathrm{b}\mathrm{s}}_{\mathrm{C}\mathrm{o}\mathrm{n}\mathrm{t}\mathrm{r}\mathrm{o}\mathrm{l}}-{\mathrm{A}\mathrm{b}\mathrm{s}}_{\mathrm{S}\mathrm{a}\mathrm{m}\mathrm{p}\mathrm{l}\mathrm{e}})/{\mathrm{A}\mathrm{b}\mathrm{s}}_{\mathrm{C}\mathrm{o}\mathrm{n}\mathrm{t}\mathrm{r}\mathrm{o}\mathrm{l}} \mathrm{x} 100$$

At physiological pH, nitric oxide generated from aqueous sodium nitroprusside (SNP) solution interacts with oxygen to produce nitrite ions, which may be quantified by the Griess Illosvoy reaction^45^. The pink chromophore generated during the diazotization of nitrite ions with sulphanilamide and subsequent coupling with NED was measured spectrophotometrically at 540 nm. Curcumin was used as a reference standard.

This assay measures the potential capacity of the plant extract to sequester metal ions. The reaction mixture contained plant extract and FeCl₂; the inhibition of the formation of the Ferrozine-Fe2⁺ complex was measured at 562 nm^46^. EDTA was used as a standard synthetic chelator for comparative calibration of the tissue’s natural chelating potential. The inhibition percentage (I %) was calculated as radical scavenging activity as follows:

$$I \left( \% \right)=( {\mathrm{A}\mathrm{b}\mathrm{s}}_{\mathrm{C}\mathrm{o}\mathrm{n}\mathrm{t}\mathrm{r}\mathrm{o}\mathrm{l}}-{\mathrm{A}\mathrm{b}\mathrm{s}}_{\mathrm{S}\mathrm{a}\mathrm{m}\mathrm{p}\mathrm{l}\mathrm{e}} )/{\mathrm{A}\mathrm{b}\mathrm{s}}_{\mathrm{C}\mathrm{o}\mathrm{n}\mathrm{t}\mathrm{r}\mathrm{o}\mathrm{l}} \mathrm{x} 100$$

The lipid peroxidation inhibition percent was assayed^47^. The absorbance was recorded at A_**532**_ nm of the organic upper layer. The inhibition percentage (I%) was calculated as radical scavenging activity as follows:

$$I \left( \% \right)=( {\mathrm{A}\mathrm{b}\mathrm{s}}_{\mathrm{C}\mathrm{o}\mathrm{n}\mathrm{t}\mathrm{r}\mathrm{o}\mathrm{l}}-{\mathrm{A}\mathrm{b}\mathrm{s}}_{\mathrm{S}\mathrm{a}\mathrm{m}\mathrm{p}\mathrm{l}\mathrm{e}} )/{\mathrm{A}\mathrm{b}\mathrm{s}}_{\mathrm{C}\mathrm{o}\mathrm{n}\mathrm{t}\mathrm{r}\mathrm{o}\mathrm{l}} \mathrm{x} 100$$

### Soluble metabolites

The content of soluble carbohydrates was assayed^48^. The developed blue-green color was read at 620 nm. Soluble carbohydrates were calculated as mg g^-1^ FW using glucose to prepare the calibration curve. The content of free amino acids was assayed^49^. Free amino acid content was calculated as mg g ^-1^ F.Wt. Using glycine to prepare a calibration curve. The content of soluble protein was assayed by the Folin reagent method^50^. Soluble proteins were calculated as mg BSA g^-1^ F.Wt. using bovine serum albumin (BSA) to prepare a calibration curve.

### Computational modeling for ZnO, α-lipoic acid, sodium nitroprusside and molecular interaction analysis

To understand the interaction of ZnO with redox-active species, such as LA and SNP, density functional theory (DFT) calculations were performed using the Gaussian 16 program suite^51^. The geometries of the ZnO unit, isolated molecules (LA and SNP), and complexes were fully optimized in the ground state using the PBE0 hybrid functional^52^ with Grimme’s dispersion correction^53^ to include long-range forces. The def2-SVP basis set^54^ was used for the applied to all atomic constituents. Numerical integrations were performed using a fine integration grid with tight self-consistent field (SCF) convergence criteria to ensure the accuracy of the results.

To obtain the most stable configurations, several possible adsorption geometries were explored during the optimization procedure. The structures with the lowest total energies were selected as the most stable conformations. Subsequently, vibrational frequency calculations were carried out at the same level of theory to confirm that the optimized geometries correspond to true minima on the potential energy surface, as indicated by the absence of imaginary frequencies.

The adsorption energy (E_ads_) of LA and SNP on ZnO was evaluated according to:

$$E_{ads} = E_{ZnO - compound} - (E_{ZnO} + E_{compound} )$$

where ${E}_{{\mathrm{ZnO}}-{\mathrm{compound}}}$ represents the total energy of the optimized ZnO–compound complex, while ${E}_{\mathrm{ZnO}}$ and ${E}_{\mathrm{compound}}$ correspond to the energies of the isolated ZnO surface and the free molecule, respectively. Negative values of ${E}_{\mathrm{ads}}$ indicate energetically favorable adsorption.

To further analyze the electronic properties of the systems, frontier molecular orbital (FMO) calculations were performed to determine the highest occupied molecular orbital (HOMO) and lowest unoccupied molecular orbital (LUMO) distributions, as well as the HOMO–LUMO energy gap. These parameters provide valuable information regarding the electronic structure, chemical reactivity, and possible charge-transfer interactions between ZnO and the adsorbed molecules.

In addition, molecular electrostatic potential (MEP) maps were generated using the Multiwfn program^55^ to identify regions of electrophilic and nucleophilic regions. Furthermore, global reactivity descriptors were computed, including chemical hardness (η), electronegativity (χ), and the electrophilicity index (ω), using the frontier molecular orbital energies and the formulas provided by Gad El-Hak et al. (2019)^56^.

### Statistical analysis

The experiment was conducted using a completely randomized design (CRD). Data were collected from three biological replicates representing six measurements derived from two independent experimental runs. Primary statistical analysis was performed using the SPSS software package (version 26.0) to determine significant differences. Mean comparisons were executed using Duncan’s Multiple Range Test (DMRT) as a post-hoc test at a significance level of *P* < 0.05. For multivariate analysis, the R software (RStudio, version. 4.2.0) was employed. Principal Component Analysis (PCA) was performed to evaluate the variance and regression ordination of all assessed attributes. The Heatmap and Scatter plots were generated using the ‘ggplot2’ and ‘corrplot’ packages. Mean values were normalized to a range of ±1 to ensure data consistency.

## Results

### Growth parameters and the allocation of biomass

**Fig. 1:**
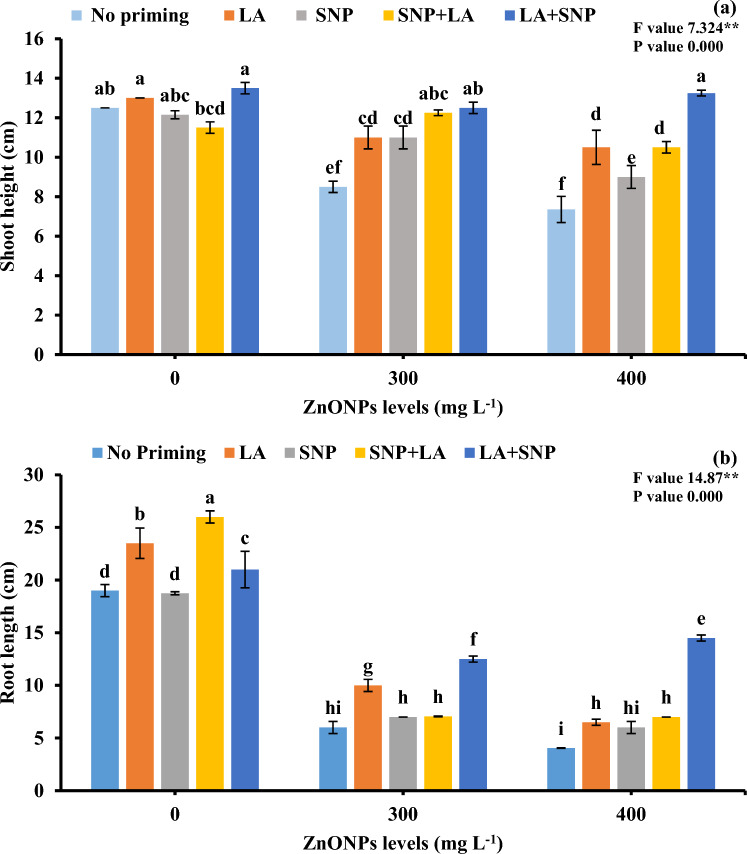
Shoot height (**a**) and root length (**b**) of [ZnO]NPs (300 and 400 mg L⁻¹) stressed wheat (*Triticum aestivum* L.) plants as affected by LA (2 mM Lipoic Acid), SNP (0.5 mM Sodium Nitroprusside), and the successive sequences priming (LA→SNP and SNP→LA) with 24h intervals. Values represent means of six replicates from two independent experiments, and vertical bars indicate ± SE. Different letters above columns indicate significant differences between treatments according to ANOVA followed by Duncan’s test at P < 0.05 level. F and P values for interaction: ** high significant, * significant, NS non-significant.

**Fig. 2:**
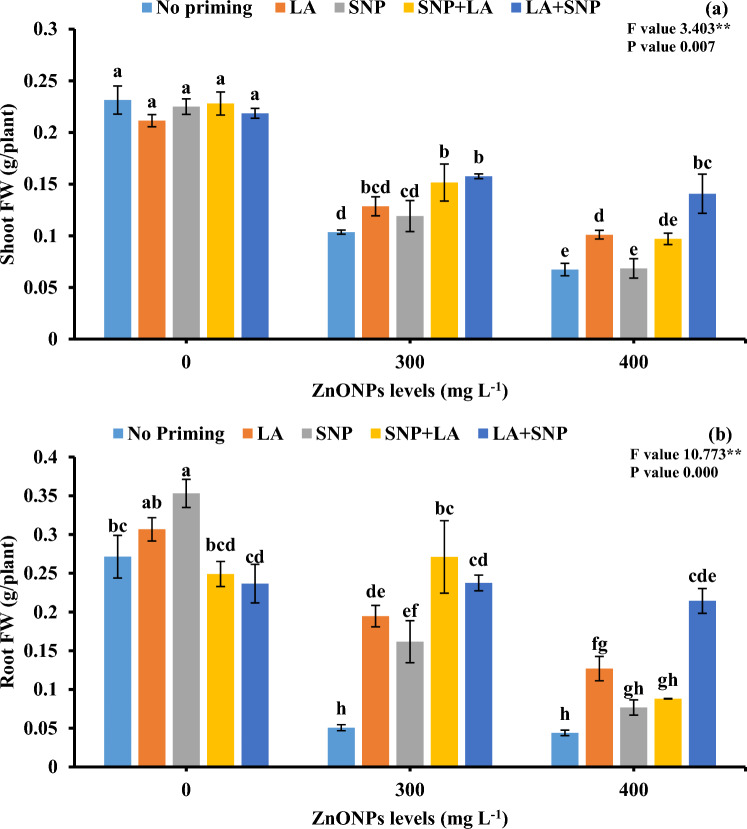
Shoot fresh weight (FW) (**a**) and root FW (**b**) of [ZnO]NPs (300 and 400 mg L⁻¹) stressed wheat (*Triticum aestivum* L.) plants as affected by LA (2 mM Lipoic Acid), SNP (0.5 mM Sodium Nitroprusside), and the successive sequences priming (LA→SNP and SNP→LA) with 24h intervals. Values represent means of six replicates from two independent experiments, and vertical bars indicate ± SE. Different letters above columns indicate significant differences between treatments according to ANOVA followed by Duncan’s test at P < 0.05 level. F and P values for interaction: ** high significant, * significant, NS non-significant.

**Fig. 3:**
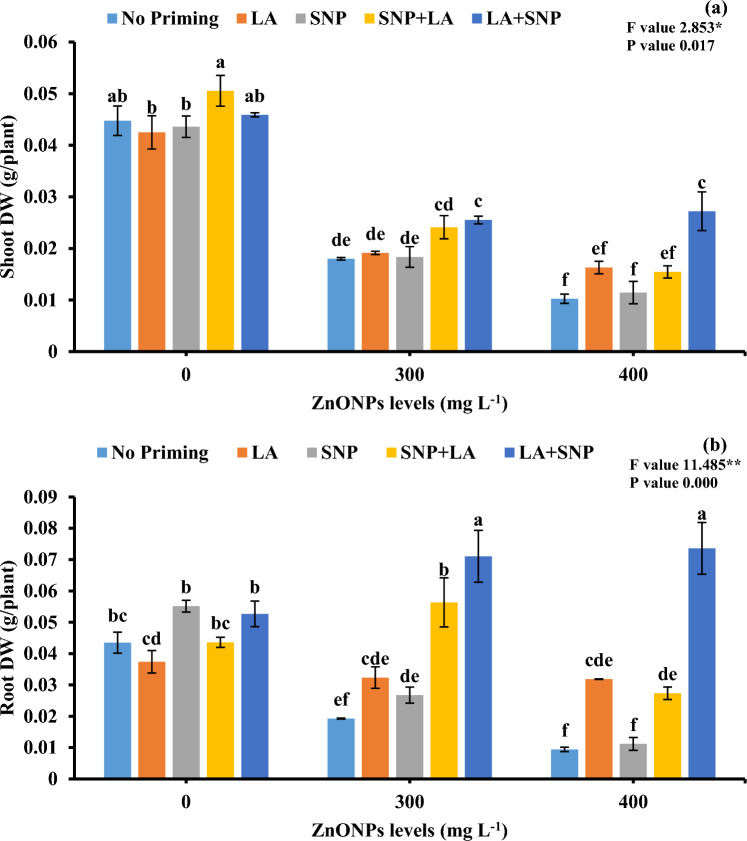
Shoot dry weight (DW) (**a**) and root DW (**b**) of [ZnO]NPs (300 and 400 mg L⁻¹) stressed wheat (*Triticum aestivum* L.) plants as affected by LA (2 mM Lipoic Acid), SNP (0.5 mM Sodium Nitroprusside), and the successive sequences priming (LA→SNP and SNP→LA) with 24h intervals. Values represent means of six replicates from two independent experiments, and vertical bars indicate ± SE. Different letters above columns indicate significant differences between treatments according to ANOVA followed by Duncan’s test at P < 0.05 level. F and P values for interaction: ** high significant, * significant, NS non-significant.

Exposure to [ZnO]NPs at (300 and 400 mg L⁻1) concentrations, retarded significantly wheat seedlings vegetative growth. Shoot height and root length as compared to control (Fig. 1a, b). This suppression in growth was affirmed by substantial reductions in fresh and dry biomass allocation in shoots and roots (Fig. 2a, b and Fig. 3a, b).

**Fig. 4:**
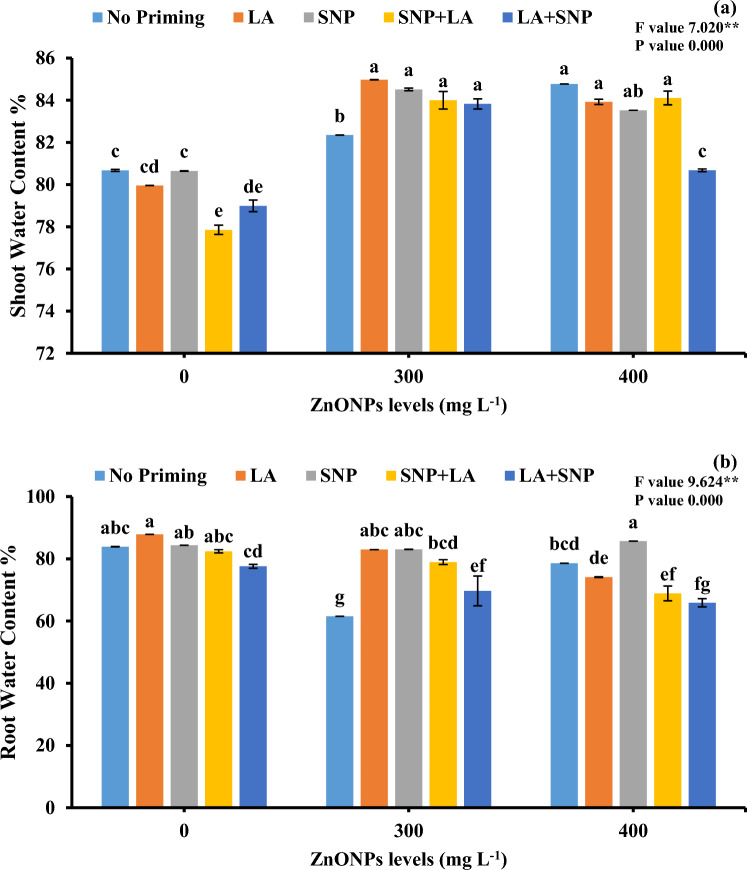
Shoot water content percentage (**a**) and root water content percentage (**b**) of [ZnO]NPs (300 and 400 mg L⁻¹) stressed wheat (*Triticum aestivum* L.) plants as affected by LA (2 mM Lipoic Acid), SNP (0.5 mM Sodium Nitroprusside), and the successive sequences priming (LA→SNP and SNP→LA) with 24h intervals. Values represent means of six replicates from two independent experiments, and vertical bars indicate ± SE. Different letters above columns indicate significant differences between treatments according to ANOVA followed by Duncan’s test at P < 0.05 level. F and P values for interaction: ** high significant, * significant, NS non-significant.

The water status showed that the root and shoot systems were affected in different ways. Significant increases in shoot water content were observed in [ZnO]NPs -treated seedlings (Fig. 4a&b), indicating root functionality and impaired water uptake under [ZnO]NPs phytotoxicity. In contrast, root water content drastically decreased.

The success of all priming agents in improving shoot height and root length corresponding to unprimed stressed plants was highly pronounce, and followed this descending rank as LA < SNP ≤ SNP+LA < LA+SNP. Successive priming with LA+SNP emerged as the most efficient treatment in order to restoring growth parameters close to, or exceeding, control under [ZnO]NPs phytotoxicity. Data presented in Fig. 3b, indicated that LA+SNP priming effectively alleviated growth retardation, with root dry mass recovering to levels comparable to the control in root dry mass by LA+SNP priming treatment, exhibiting a remarkable recovery, reaching values significantly higher than corresponding unprimed stressed treatment and, in some cases, surpassing the control.

For all growth-related parameters, a highly significant statistical interaction between [ZnO]NPs concentration and priming treatments was found, highlighting the crucial role of priming—especially successive LA+SNP application—in reprogramming growth responses under ZnO nanoparticle stress.

### The integrity of photosynthetic pigment

**Fig. 5:**
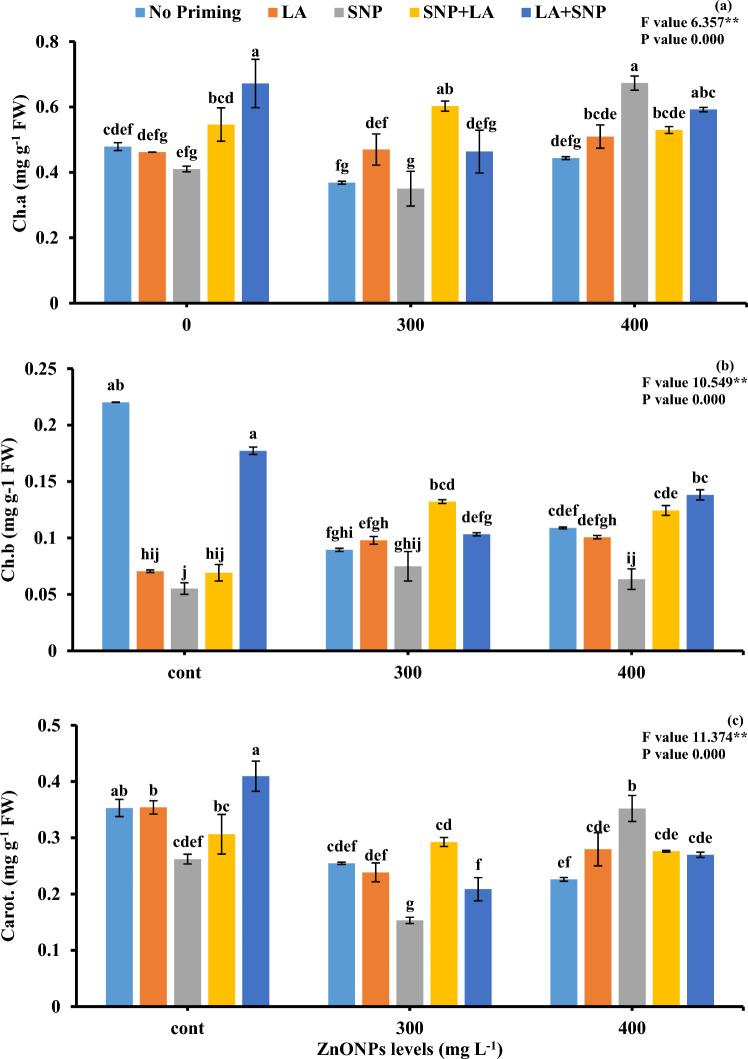
Photosynthetic pigments, Chlorophyll a Ch.a (**a**) chlorophyll b Ch.b (**b**) and carotenoids (5C) of [ZnO]NPs (300 and 400 mg L⁻¹) stressed wheat (*Triticum aestivum* L.) plants as affected by LA (2 mM Lipoic Acid), SNP (0.5 mM Sodium Nitroprusside), and the successive sequences priming (LA→SNP and SNP→LA) with 24h intervals. Values represent means of six replicates from two independent experiments, and vertical bars indicate ± SE. Different letters above columns indicate significant differences between treatments according to ANOVA followed by Duncan’s test at P < 0.05 level. F and P values for interaction: ** high significant, * significant, NS non-significant.

The composition of photosynthetic pigment was severely disrupted due to the exposure of wheat seedlings to [ZnO]NPs phytotoxicity. The contents of chlorophyll a, chlorophyll b, and carotenoid were declined significantly, with an obvious dose-dependent reduction (300 > 400 mg L⁻1) (Fig. 5a–c). These observations reflected the substantial disfunction to the photosynthetic apparatus under [ZnO]NPs -induced oxidative stress.

Pigment loss was markedly buffered due to Seed priming. Successive LA+SNP priming treatment conferred generally the highest protective effect, while chlorophyll a content reached its highest level and restored its content under LA+SNP-treatment (Fig. 5a). This trend was reobserved for chlorophyll b, where LA+SNP priming sustained pigment content closest to those of the control, (Fig. 5b). Carotenoid contents exhibited sensitive response to [ZnO]NPs phytotoxicity, all priming treatments counteracted partially their decline, with LA+SNP again showing the most effective sustention (Fig. 5c).

### Soluble metabolites modulation

**Fig. 6:**
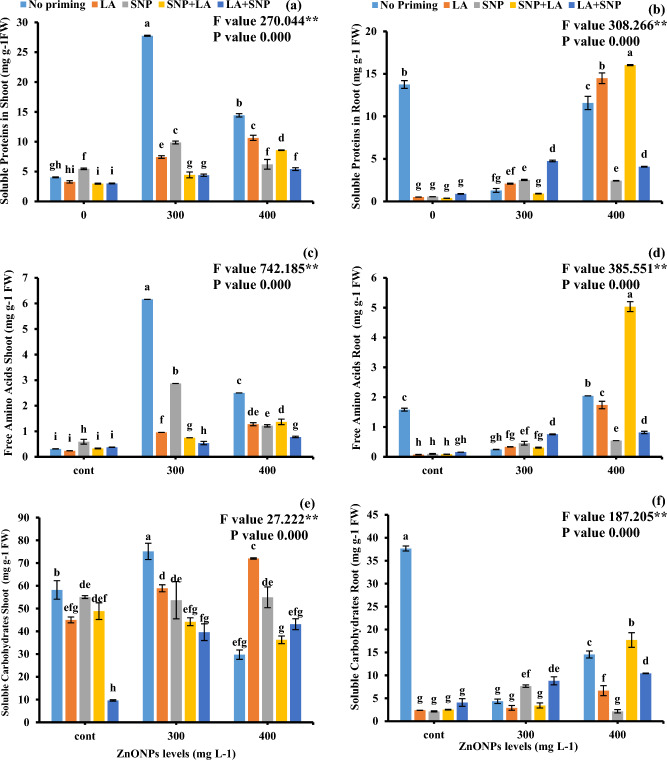
Soluble metabolities (**a**,**b**) soluble proteins (**c**,**d**) Free amino acids and (**e**,**f**) soluble carbohydrates of [ZnO]NPs (300 and 400 mg L⁻¹) stressed wheat (*Triticum aestivum* L.) plants as affected by LA (2 mM Lipoic Acid), SNP (0.5 mM Sodium Nitroprusside), and the successive sequences priming (LA→SNP and SNP→LA) with 24h intervals. Values represent means of six replicates from two independent experiments, and vertical bars indicate ± SE. Differentletters above columns indicate significant differences between treatments according to ANOVA followed by Duncan’s test at P < 0.05 level. F and P values for interaction: ** high significant, * significant, NS non-significant.

Metabolic homeostasis was altered significantly in wheat seedlings by activating soluble osmolytes accumulation due to [ZnO]NPs phytotoxicity. The contents of soluble protein increased markedly in shoots and roots under [ZnO]NPs treatments, particularly at 400 mg L⁻^1^ (Fig. 6a, b). The application of most seed priming treatments further increased protein content, underscoring the accumulation of functional and stress-responsive proteins accumulation.

The pattern of free amino acids generally was similar to soluble proteins, with lower absolute values, supposing its supportive role as potent compatible solutes during stress adaptation (Fig. 6c, d). Likewise, soluble carbohydrates showed significant elevated values in shoots under [ZnO]NPs stress and further accumulated by priming treatments (Fig. 6e, f).

Among all priming techniques, LA+SNP successive application enhanced consistently the highest soluble metabolites content, implying to its effective role in reinforcing metabolic buffering and osmotic adjustment to counteract physiological strain due to [ZnO]NPs -induced exposure.

### Antioxidant machinery induction

**Fig. 7:**
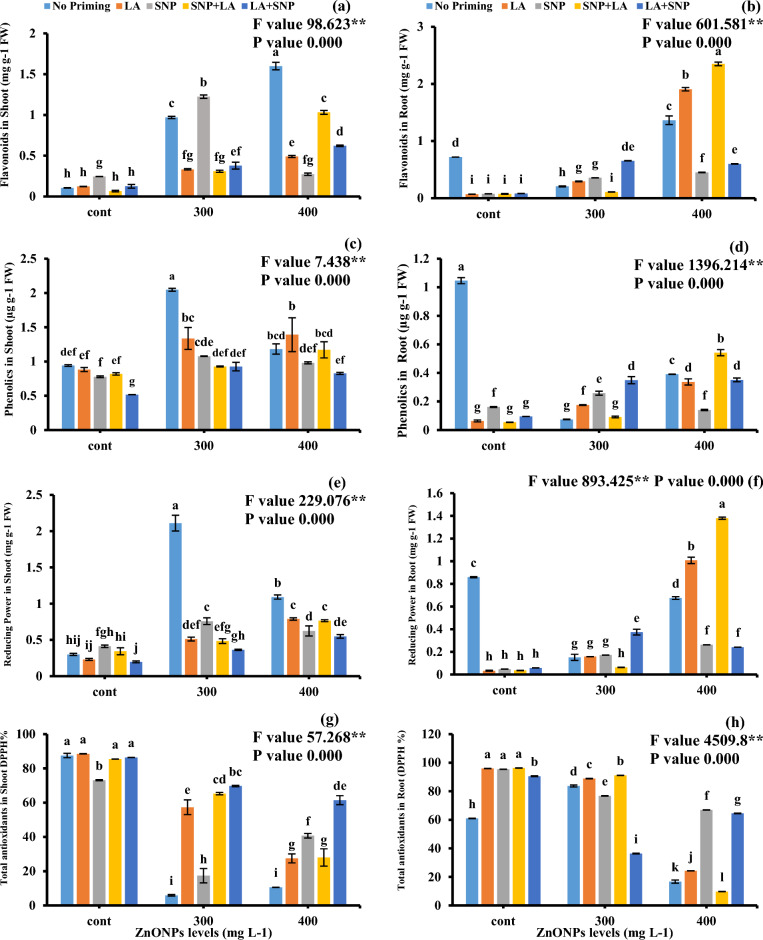
Non-enzymatic antioxidants (**a**,**b**) Flavonoids, (**c**,**d**) phenolics, (**e**,**f**) reducing power and (**g**,**h**) total antioxidants of [ZnO]NPs (300 and 400 mg L⁻¹) stressed wheat (*Triticum aestivum* L.) plants as affected by LA (2 mM Lipoic Acid), SNP (0.5 mM Sodium Nitroprusside), and the successive sequences priming (LA→SNP and SNP→LA) with 24h intervals. Values represent means of six replicates from two independent experiments, and vertical bars indicate ± SE. Different letters above columns indicate significant differences between treatments according to ANOVAfollowed by Duncan’s test at P < 0.05 level. F and P values for interaction: ** high significant, * significant, NS non-significant.

Enzymatic and non-enzymatic antioxidant systems were obviously affected by [ZnO]NPs phytotoxicity and seed priming applications. Non-enzymatic antioxidants accumulation, including phenolics and flavonoids in shoots and roots, was significantly recorded under [ZnO]NP phytotoxicity (Fig. 7a–d). Zinc oxide nanoparticle [ZnO]NPs phytotoxicity induced significant phenolic content accumulation in shoots, whereas a reduction was noticed in roots, that was partially counteracted by priming, particularly at 300 mg L⁻1. Stressed wheat seedlings showed induced flavonoids content, while priming changed this response in a treatment and organ-specific manner.

Reducing power data exhibited varied trends between shoot and root: a significant enhancement recorded in shoots but sharp decline recorded in roots under [ZnO]NPs toxicity (Fig. 7e, f). Priming application noticeably modified these responses, restoring redox hemostasis in stressed wheat seedlings. Zinc oxide nanoparticle [ZnO]NPs phytotoxicity resulted in remarked reduction in total antioxidant capacity, while significant induction shoots and roots were recorded as a consequence of priming application (Fig. 7g, h).

**Fig. 8:**
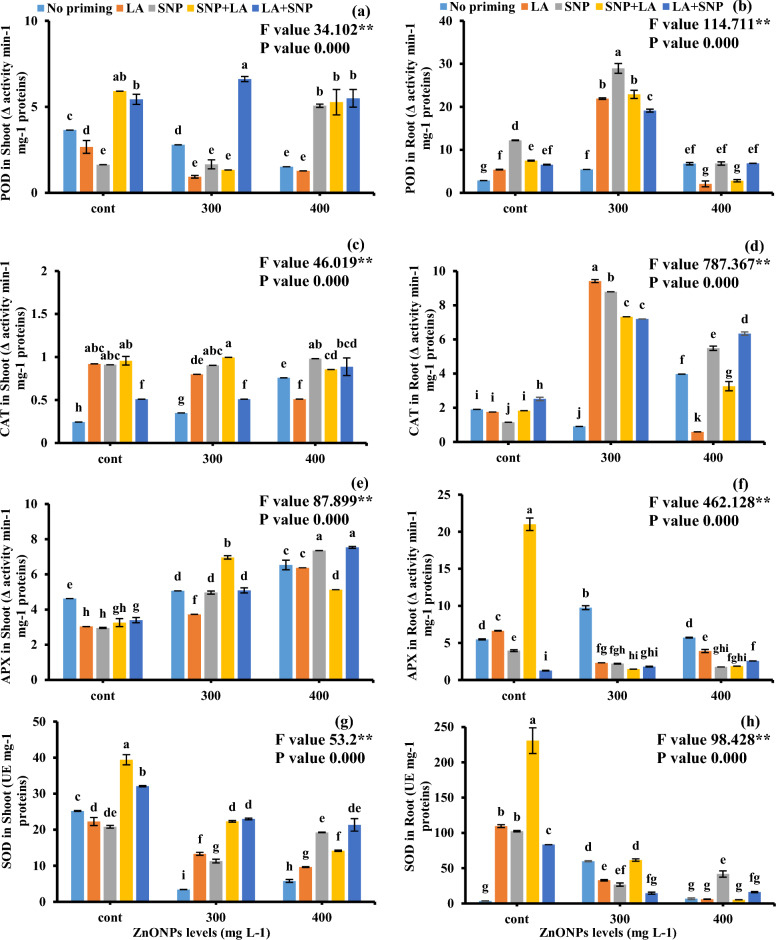
Enzymatic antioxidants (**a**,**b**) POD, (**c**,**d**) CAT, (**e**,**f**) APX and (**g**,**h**) SOD enzymes of [ZnO]NPs (300 and 400 mg L⁻¹) stressed wheat (*Triticum aestivum* L.) plants as affected by LA (2 mM Lipoic Acid), SNP (0.5 mM Sodium Nitroprusside), and the successive sequences priming (LA→SNP and SNP→LA) with 24h intervals. Values represent means of six replicates from two independent experiments, and vertical bars indicate ± SE. Different letters above columns indicate significant differences between treatments according to ANOVA followed by Duncan’s test at P < 0.05 level. F and P values for interaction: ** high significant, * significant, NS non-significant.

In respect of enzymatic antioxidants, [ZnO]NPs phytotoxicity improved intrinsic reprogramming of enzymatic antioxidants activities. The activity of peroxidase (POD) exhibited multiple organ-specific trends, with substantial POD activity enhancement due to priming under 300 mg L⁻1 [ZnO]NPs (Fig. 8a, b). Catalase (CAT) and ascorbate peroxidase (APX) activities were enhanced significantly under phytotoxicity of [ZnO]NPs and further increased by priming application, particularly at 300 mg L⁻1 [ZnO]NPs (Fig. 8c–f), implying at improved H₂O₂ detoxification capacity.Superoxide dismutase (SOD) activity sharply declined in shoots under [ZnO]NPs toxicity, but a significant restoration recorded under all priming treatments, with the strongest improvement recorded at successive priming (Fig. 8g). Contrarily, SOD activity in root ascended under [ZnO]NPs toxicity and was enhanced further by successive priming techniques (Fig. 8h). .

### Radical scavenging efficiencies and chelating ability

Zinc oxide nanoparticle [ZnO]NPs phytotoxicity resulted in a pronounced decline in the antioxidant scavenging capacities of wheat seedlings, with distinct responses depending on reactive species type, [ZnO]NPs level, priming agents and organ-specific manner (Table 1).

**Table 1:** Effect seed priming with redox agents (individually or successively) on free radical scavenging abilities of wheat plant under ZnO NPs.

Priming agents	ZnONPs mgL^-1^	OH^•-^		H_2_O_2_		NOS		Metal chelating		Lipid peroxidation	
		**%**									
		**Shoot**	**Root**	**Shoot**	**Root**	**Shoot**	**Root**	**Shoot**	**Root**	**Shoot**	**Root**
No priming	0	81.9b ±0.0	34.0h ±0.3	62.7b ±0.1	63.3e ±3.0	92.2b ±0.0	50.3h ±0.2	92.6c ±0.0	66.5d ±2.2	84.3ab ±1.2	72.2e ±0.5
	300	14.9k ±0.7	88.3c ±0.1	12.3h ±0.6	77.8d ±0.9	21.1l ±0.2	87.9d ±0.2	21.2l ±0.3	93.8b ±0.0	6.50i ±0.2	89.3bcd ±0.8
	400	19.4j ±0.9	26.8i ±0.4	24.0f ±1.1	26.2i ±0.0	30.9k ±1.4	31.2i ±1.5	54.5k ±0.2	68.1d ±0.3	25.6h ±2.2	33.3h ±4.4
LA	0	87.9a ±0.1	97.0a ±0.0	76.2a ±1.4	94.1a ±0.2	95.0a ±0.1	97.6a ±0.0	96.6a ±0.0	98.6a ±0.0	84.1ab ±2.3	96.9a ±0.4
	300	60.3ef ±0.2	87.5c ±0.1	18.7g ±3.1	75.7d ±0.0	81.8d ±0.2	88.3d ±0.4	87.9e ±0.1	93.4b ±0.0	69.2e ±0.1	84.0d ±0.6
	400	45.1h ±0.1	13.7j ±0.3	30.5e ±1.9	19.6j ±0.1	72.1f ±0.4	23.9j ±0.0	82.7g ±0.2	41.4f ±1.9	53.5f ±3.1	16.8i ±2.2
SNP	0	79.9bc ±0.0	96.8a ±0.0	55.2c ±0.3	93.7a ±0.4	85.0c ±0.3	97.4a ±0.0	92.2c ±0.0	98.6a ±0.0	81.4bc ±0.0	94.1ab ±2.0
	300	34.7i ±2.9	85.0d ±0.1	21.0fg ±1.1	65.1e ±0.0	35.2j ±0.3	84.0e ±0.0	74.6j ±0.1	94.2b ±0.1	41.1g ±0.5	73.0e ±4.2
	400	58.9f ±0.1	78.4f ±0.0	10.4h ±2.7	51.8f ±0.2	57.8i ±0.0	82.6e ±0.3	81.3h ±0.1	92.8b ±0.5	11.1i ±0.1	74.2e ±0.3
SNP +LA	0	89.7a ±0.1	97.3a ±0.0	78.5a ±0.6	94.1a ±0.0	95.0a ±0.0	97.6a ±0.0	94.4b ±0.3	99.2a ±0.0	73.3de ±2.5	97.1a ±0.7
	300	70.3d ±0.1	93.4b ±0.0	41.3d ±1.4	85.5c ±0.6	78.2e ±0.3	93.6c ±0.1	85.0f ±0.0	97.8a ±0.1	69.8e ±1.1	90.7bc ±1.4
	400	52.5g ±1.2	86.0c ±0.1	11.9h ±0.6	11.5k ±1.6	61.9h ±1.3	13.8k ±1.3	76.5i ±0.2	52.6e ±1.1	52.3f ±1.1	9.10j ±0.4
LA+ SNP	0	90.2a ±0.0	94.1b ±0.0	80.4a ±0.8	89.0b ±0.1	93.7ab ±0.4	95.2b ±0.0	94.7b ±0.2	98.1a ±0.0	88.8a ±0.9	85.6cd ±2.3
	300	79.0c ±0.5	67.5g ±1.7	57.3c ±1.9	34.4h ±0.8	84.8c ±0.9	65.7g ±0.3	89.3d ±0.5	89.4c ±0.1	78.1cd ±1.8	46.4g ±0.2
	400	61.5e ±0.2	80.9e ±0.6	19.4g ±0.4	44.5g ±0.4	67.7g ±1.1	76.9f ±0.05	82.8g ±0.6	89.1c ±0.2	57.7f ±3.7	59.8f ±0.0

Values represent means of six replicates, from two independent experiments and the vertical bars indicate ± SE. Different letters indicate significant difference between treatments according to ANOVA, Duncan’s test at P<0.05 level.

The ability to scavenge nitric oxide was highest in wheat control shoots (both primed and unprimed), but it was greatly lowered in plants that were treated with [ZnO]NPs (Table 1). Priming enhanced the nitric oxide scavenging capacity in shoots treated with [ZnO]NPs. Control roots exhibited a 50% lower nitric oxide scavenging capacity compared to shoots; however, this capacity was significantly enhanced following priming treatments. The lower concentration of [ZnO]NPs (300 mgL^-1^), regardless of priming, predominantly preserved control values, whereas the elevated concentration (400 mgL^-1^) diminished scavenging ability in comparison to control roots. Priming with SNP or SNP+LA significantly enhanced nitric oxide scavenging capacity.

The percentage of OH• scavenging in shoots and roots followed a pattern similar to that of nitric oxide scavenging (Table 1). The values of radical scavenging went down as the [ZnO]NPs concentrations went up. In control treatments, wheat shoots were best at getting rid of H_2_O_2_, but [ZnO]NPs retarded plant ability. Priming worked with 400 mg L^-1^ [ZnO]NPs but not with 300 mg L^-1^. The order was (LA+SNP < SNP+LA < SNP < LA).

All primed roots, on the other hand, showed a 20–30% increase in their ability to scavenge H_2_O_2_ under control conditions. Both lower and higher levels of [ZnO]NPs retarded plant ability for primed plants to get rid of H_2_O_2_. However, individual SNP priming at 400 mg L^-1^ [ZnO]NPs, successive SNP+LA at 300 mg L^-1^ [ZnO]NPs, and LA+SNP at 400 mg L^-1^ [ZnO]NPs enhanced H_2_O_2_ scavenging capacity relative to non-primed stressed treatments (Table 1).

In unprimed plants, [ZnO]NPs significantly reduced the ability of shoots to chelate metals (300 > 400 mgL^-1^); priming restored most of this ability (Table 1). Roots had higher percentages of metal chelation, but this was only stopped at 400 mg L^-1^ [ZnO]NPs in plants that had been primed with LA or SNP+LA.

In shoots, the ability to stop lipid peroxidation was highest in control plants (primed or unprimed) and very low in unprimed [ZnO]NPs treatments (300 > 400 mg L^-1^). All priming agents improved the ability of shoots to stop lipid peroxidation, except for SNP at 400 mg L^-1^ [ZnO]NPs. In roots, this capacity was stimulated in unprimed plants at 300 mg L^-1^ [ZnO]NPs but diminished markedly at 400 mg L^-1^ (Table 1). The root’s ability to stop lipid peroxidation at the 400 mg L^-1^ [ZnO]NPs level was restored by applying SNP alone or in a series with LA+SNP.

### PCA, hierarchical clustering, and correlation analysis

**Fig. 9:**
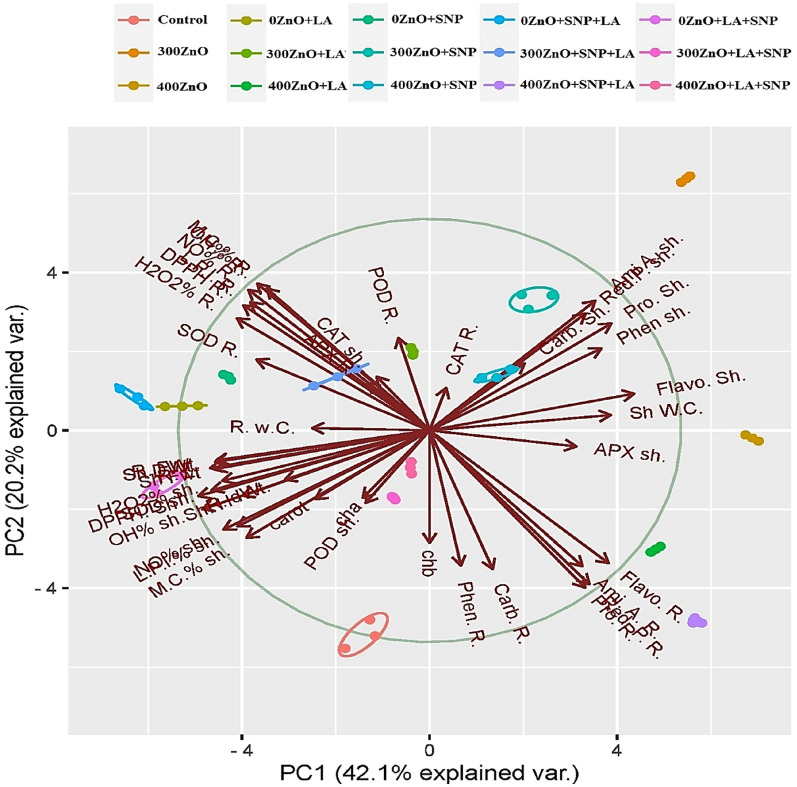
Principal component analysis (PCA) of the studied parameters in shoot and root of wheat plants with priming agents LA (2 mM Lipoic Acid), SNP (0.5 mM Sodium Nitroprusside), and the successive sequences priming (LA→SNP and SNP→LA) with 24h intervals. under different levels of ZnONPs (0, 300 and 400 mg L^-1^) on the studied parameters in shoot and root of wheat plants. SH.H shoot height; R.L. root length; ShFwt and ShDwt, shoot Fresh and Dry weight; Chl a, Chlorophyll a; Chl b, Chlorophyll b; Carot. carotenoids; Sh.W.C. shoot water content; R.W.C. root water content; pro. Sh. Shoot Proteins; Ami.A.Sh amino acids shoot; Carb.sh, carbohydrates in shoot; Red.P.Sh, reducing power shoot; APX, Ascorbate peroxidase, CAT, Catalase; POD, Peroxidase; SOD, Superoxide dismutase; Phen, Free phenolic; Flav, total flavonoids; L.P.I Inhibition of lipid peroxide formation %; H_2_O_2_, hydrogen peroxide scavenging %; DPPH, Total antioxidant activity; OH, Hydroxyl radical (OH^.-^) scavenging %; NO, Nitric oxide scavenging %; M. C, Metal chelating %.

The PCA biplot (Fig. 9), which used 43 physiological variables and 15 treatments, showed that the first two principal components explained 42.1% (Axis 1) and 20.2% (Axis 2) of the variance. The analysis showed a difference between the shoot water content, soluble metabolites (carbohydrates, proteins, amino acids), flavonoids, phenolics, and reducing power (on the right side) and all antioxidant abilities, chlorophyll a, root water content, and growth indicators (on the left side). The right-hand half was greatly affected by 300 and 400 mg L^-1^ [ZnO]NPs, 300 and 400 mg L^-1^ [ZnO]NPs + SNP, 400 mg L^-1^ [ZnO]NPs + LA, and 400 mg L^-1^ [ZnO]NPs + (SNP, LA). The left side was mostly affected by control treatments, all three levels of [ZnO]NPs with LA+SNP successive priming, and the 0 and 300 mg L^-1^ levels with either LA or SNP+LA successive priming.

**Fig. 10:**
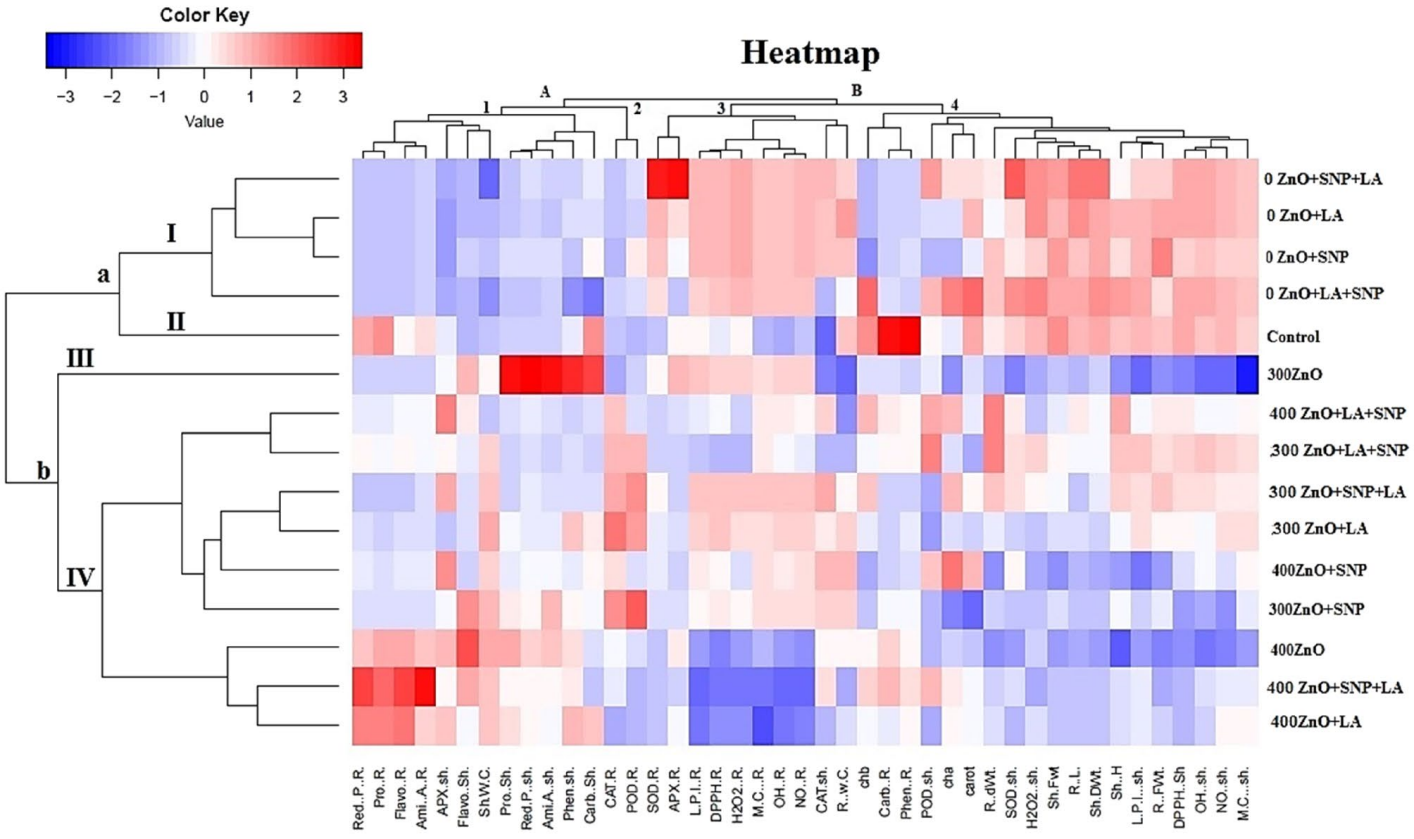
Heatmap showing the saturation of colors indicating effects of priming agents LA (2 mM Lipoic Acid), SNP (0.5 mM Sodium Nitroprusside), and the successive sequences priming (LA→SNP and SNP→LA) with 24h intervals. under different levels of ZnONPs (0, 300 and 400 mg L^-1^) on the studied parameters in shoot and root of wheat plants. SH.H shoot height; R.L. root length; ShFwt and ShDwt, shoot Fresh and Dry weight; Chl a, Chlorophyll a; Chl b, Chlorophyll b; Carot. carotenoids; Sh.W.C. shoot water content; R.W.C. root water content; pro. Sh. Shoot Proteins; Ami.A.Sh amino acids shoot; Carb.sh, carbohydrates in shoot; Red.P.Sh, reducing power shoot; APX, Ascorbate peroxidase, CAT, Catalase; POD, Peroxidase; SOD, Superoxide dismutase; Phen, Free phenolic; Flav, total flavonoids; L.P.I Inhibition of lipid peroxide formation %; H_2_O_2_, hydrogen peroxide scavenging %; DPPH, Total antioxidant activity; OH, Hydroxyl radical (OH^.-^) scavenging %; NO, Nitric oxide scavenging %; M. C, Metal chelating %.

The two-sided dendrogram and heatmap (Fig. 10), put growth traits with antioxidant enzymes (CAT, POD, and SOD), antioxidant abilities (metal chelating, NO scavenging, H_2_O_2_ scavenging, OH• scavenging, lipid peroxidation inhibition), and DPPH in subcluster A. Subcluster B brought together soluble metabolites (like proteins, amino acids, and carbohydrates from shoots) with flavonoids and reducing power.

**Fig. 11:**
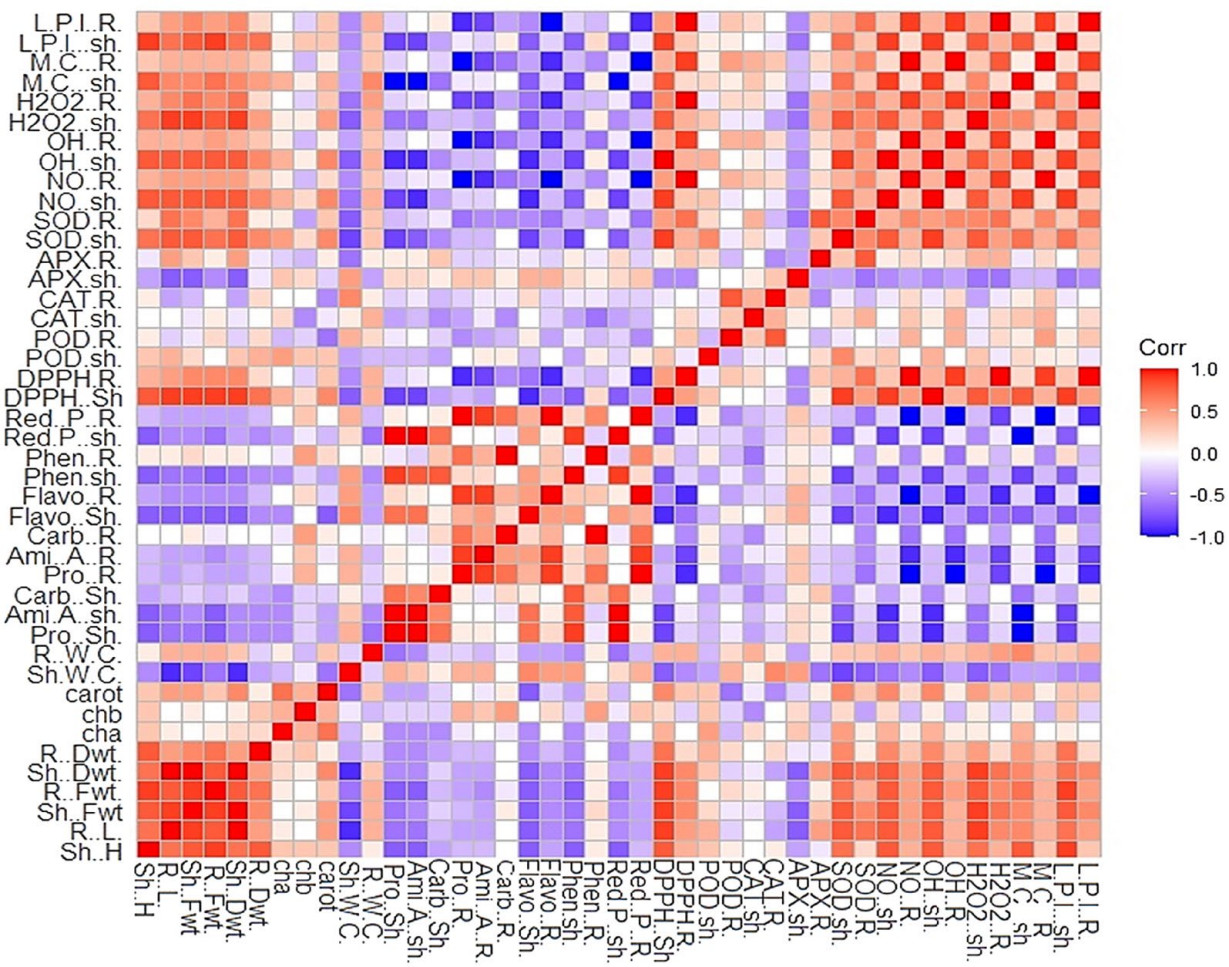
Correlation matrix of the 28 measured traits under priming agents LA (2 mM Lipoic Acid), SNP (0.5 mM Sodium Nitroprusside), and the successive sequences priming (LA→SNP and SNP→LA) with 24h intervals. under different levels of ZnONPs (0, 300 and 400 mg L^-1^) on the studied parameters in shoot and root of wheat plants. The increasing color intensities illustrate a higher correlation coefficient. SH.H shoot height; R.L. root length; ShFwt and ShDwt, shoot Fresh and Dry weight; Chl a, Chlorophyll a; Chl b, Chlorophyll b; Carot. carotenoids; Sh.W.C. shoot water content; R.W.C. root water content; pro. Sh. Shoot Proteins; Ami.A.Sh amino acids shoot; Carb.sh, carbohydrates in shoot; Red.P.Sh, reducing power shoot; APX, Ascorbate peroxidase, CAT, Catalase; POD, Peroxidase; SOD, Superoxide dismutase; Phen, Free phenolic; Flav, total flavonoids; L.P.I Inhibition of lipid peroxide formation %; H_2_O_2_, hydrogen peroxide scavenging %; DPPH, Total antioxidant activity; OH, Hydroxyl radical (OH^.-^) scavenging %; NO, Nitric oxide scavenging %; M. C, Metal chelating %.

Correlation analysis revealed significant negative correlations between soluble metabolites, phenolics, flavonoids, and reducing power, contrasted with all antioxidant capacities and DPPH. On the other hand, there was a strong positive relationship between these antioxidant parameters and growth traits in both shoots and roots (Fig. 11).

### Simulated calculations

#### Electronic structure and FMO analysis

**Fig. 12:**
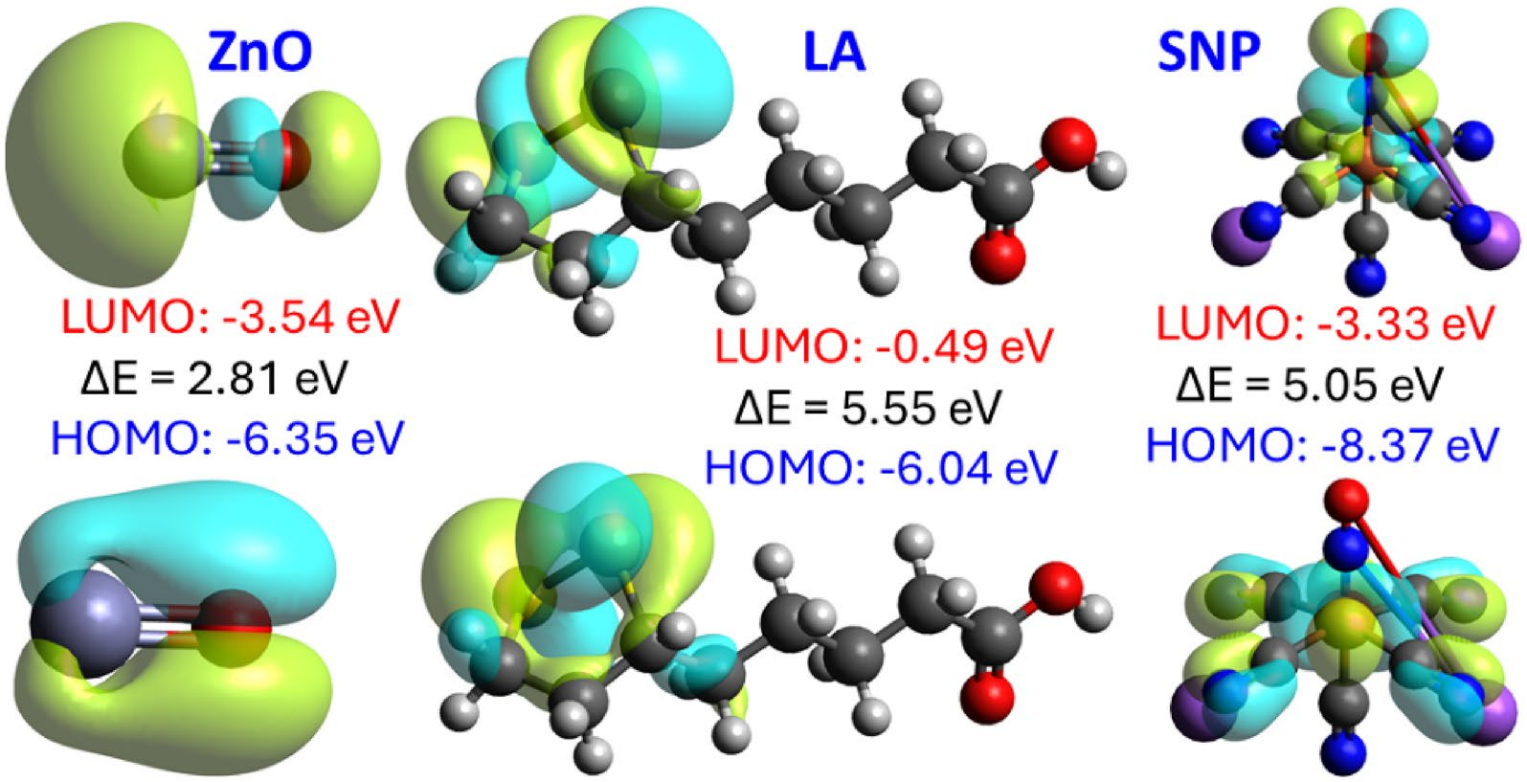
Frontier molecular orbital (HOMO and LUMO) isosurface distributions of ZnO, α-lipoic acid (LA), and sodium nitroprusside (SNP), along with their corresponding orbital energies, calculated at the PBE0-D3BJ/def2-SVP level of theory.

Geometric optimizations were performed for ZnO, α-lipoic acid (LA), and sodium nitroprusside (SNP), and their respective electronic properties were computed using the PBE0-D3BJ/def2-SVP method. The frontier molecular orbitals, as illustrated in figure 12, indicated that the HOMO for ZnO is positioned over the Zn-O bond and has a π-type character, while the LUMO has antibonding σ* character. The HOMO-LUMO gaps (ΔE_g_), computed for the molecules, were 2.81 eV for ZnO, 5.55 eV for LA, and 5.05 eV for SNP (Table 2). The reduced energy gap in ZnO implies increased chemical reactivity and a potential for enhanced charge-transfer interactions with LA and SNP. Fig. 12

**Table 2:** Global reactivity descriptors of ZnO, α-lipoic acid (LA), and sodium nitroprusside (SNP) calculated at the PBE0-D3BJ/def2-SVP level of theory. ΔE_g_ represents the HOMO–LUMO energy gap, η the chemical hardness, χ the electronegativity, and ω the electrophilicity index. Dipole moments are reported in Debye, while all other quantities are given in eV.

**Comp.**	**Dipole**	**HOMO**	**LUMO**	**ΔE**_**g**_	$\eta$	$\chi$	**ω**
**ZnO**	**5.58**	**-6.35**	**-3.54**	**2.81**	**1.41**	**4.95**	**8.70**
**LA**	**2.25**	**-6.04**	**-0.49**	**5.55**	**2.77**	**3.26**	**1.92**
**SNP**	**3.08**	**-8.37**	**-3.33**	**5.05**	**2.52**	**5.85**	**6.78**

#### Molecular electrostatic potential (MEP) mapping

**Fig. 13:**
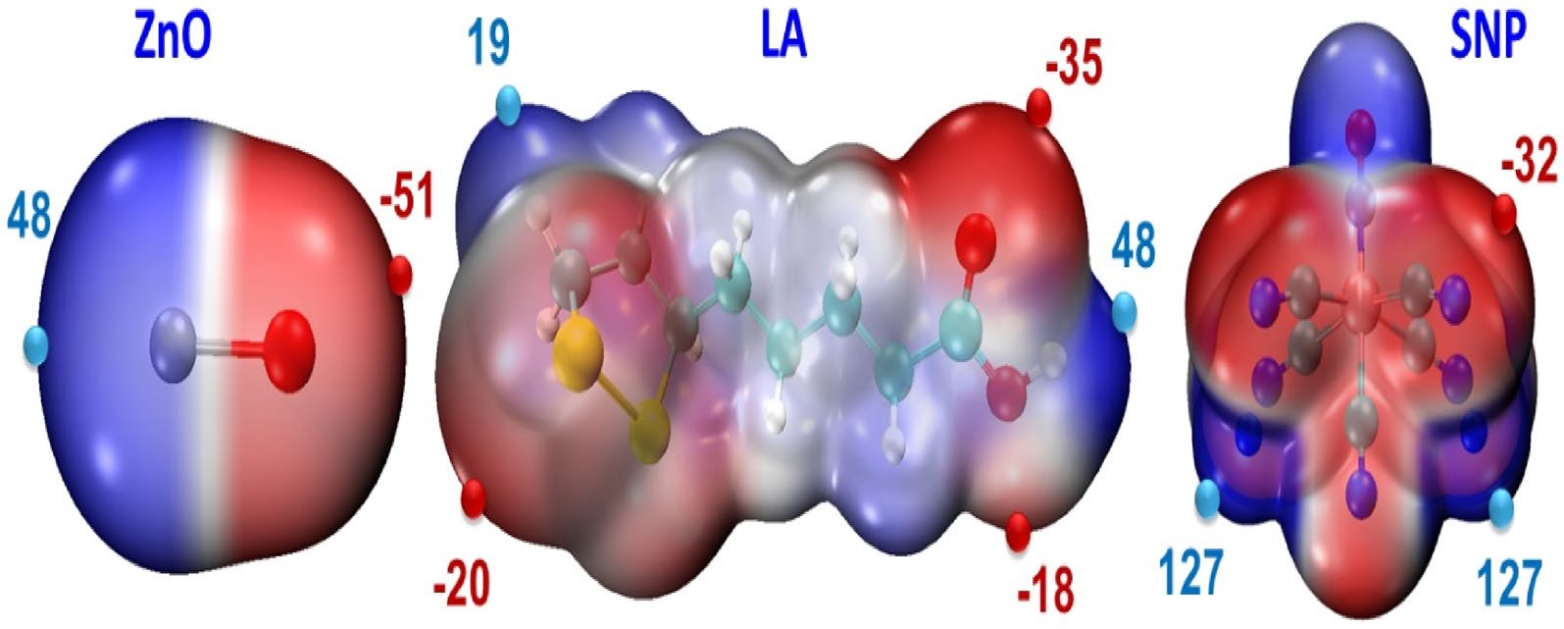
Molecular electrostatic potential (MEP) surface maps of ZnO, α-lipoic acid (LA), and sodium nitroprusside (SNP), showing the electrostatic potential maxima and minima (in kcal·mol⁻¹), calculated at the PBE0-D3BJ/def2-SVP level of theory.

Figure 13 illustrates the MEP mapping for the molecules. The MEP mapping for ZnO and LA shows negative potentials for the oxygen atoms, at −51 and −35 kcal·mol⁻^1^, respectively. In the case of SNP, the negative potentials are localized over the cyanide ligands at −32 kcal·mol⁻^1^. In contrast, the Zn atom in ZnO and the hydrogen atom bonded to the carboxyl oxygen in LA show high positive potentials at +48 kcal·mol⁻^1^. Notably, the sodium cation in SNP shows the highest positive potential at +127 kcal·mol⁻^1^Fig. 13.

#### Adsorption geometries and energies

**Fig. 14:**
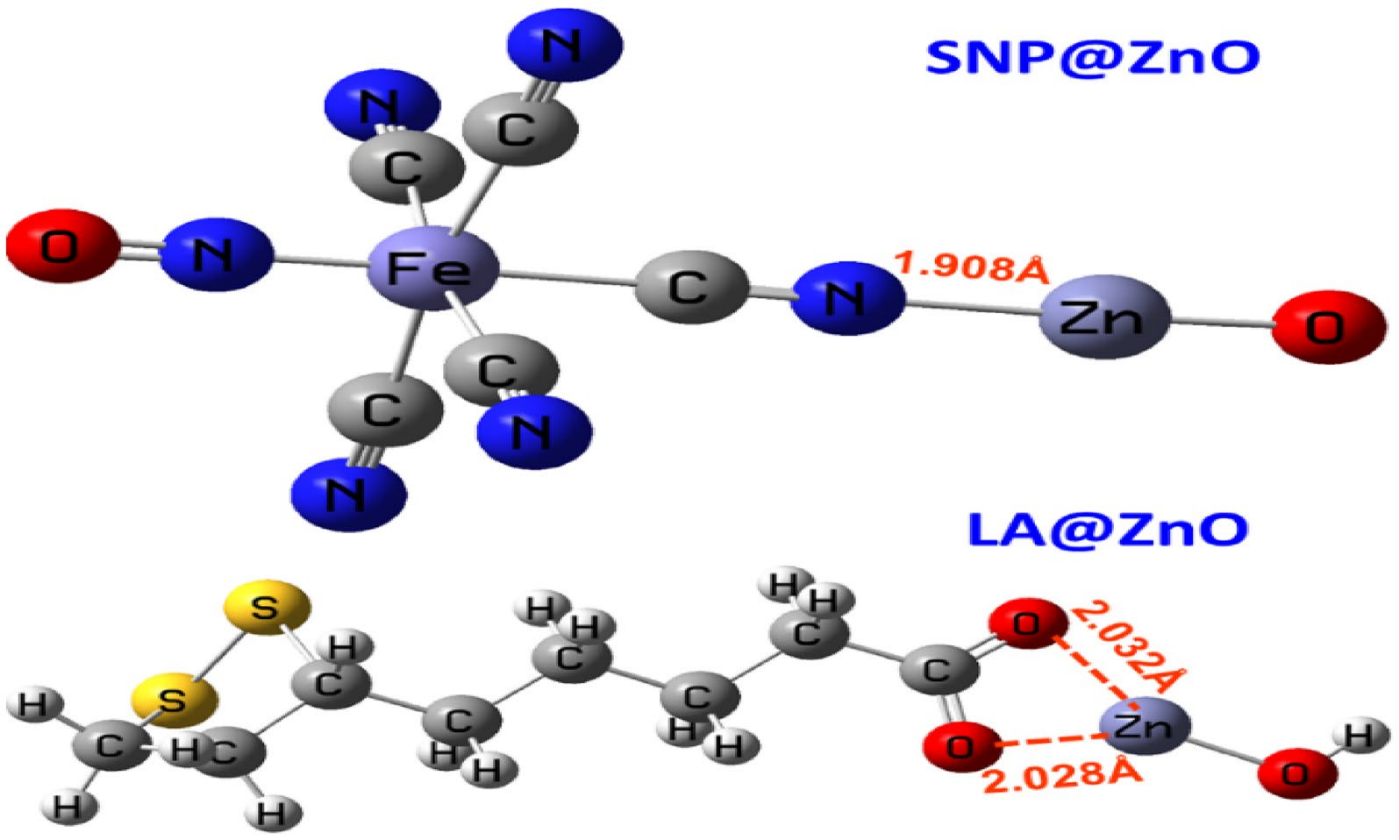
The geometry of the adsorbed α-lipoic acid (LA) and sodium nitroprusside (SNP) on the surface of ZnO calculated at the PBE0-D3BJ/def2-SVP level of theory.

Figure 14 depicts the optimized adsorption geometries for the complexes. In the ZnO-LA complex, a proton transfer from the carboxyl group to the ZnO moiety is observed, resulting in a bidentate coordination mode for LA with the Zn center through two oxygen atoms at a Zn...O separation of 2.03 Å. This interaction stretches the Zn=O bond from 1.694 Å to 1.781 Å. In the ZnO-SNP complex, the Zn center forms a coordination bond with the nitrogen atom of one of the cyanide ligands at a Zn...N separation of 1.908 Å. This interaction causes a slight shortening of the Zn=O bond from 1.694 Å to 1.683 Å. The adsorption energies (E_ads_) were computed to be −118.1 kcal·mol⁻^1^ for the LA complex and −110.1 kcal·mol⁻^1^ for the SNP complexFig. 14.

## Discussion

The present study elucidates the complex responses of wheat seedlings to [ZnO]NPs stress. The observed reduction in shoot height and root length indicates acute phytotoxicity at the concentrations used (300–400 mg L⁻^1^), which aligns with recent findings that high concentrations of [ZnO]NPs induce severe cellular damage in cereal crops^57,58^. Interestingly, while our previous study^30^ demonstrated that ZnO at a lower dose (60 mg L^-1^) serves as a biofortifying agent that enhances drought tolerance, the current findings highlight a toxic threshold where high-dose nano-exposure disrupts primary growth. This shift from biofortification to toxicity underscores the dose-dependent nature of nanomaterials. The observed shoot succulence suggests a “Dilution Mechanism”a strategic physiological response to decrease the internal concentration of penetrating nano-ions, a phenomenon also reported in plants facing heavy metal and salt stress to maintain cellular hydration^59,60^.

To counteract [ZnO]NPs-induced damage, the successive seed priming technique (LA+SNP) was employed—a novel approach in seed technology. The superiority of this sequential treatment is consistent with our recent work^31^, which established that sequential seed priming (e.g., SNP + CaCl_2_) significantly outperforms individual treatments by orchestrating a more robust early ROS detoxification. While LA and SNP individually act as sources of H_2_S and NO signaling, their alternate successive application (LA+SNP) creates a Double-layered defense. This synergy likely triggers a priming memory and a signaling crosstalk between NO and H_2_S that prepares the embryo more effectively for severe environmental constraints^61–64^.

The degradation of photosynthetic pigments by [ZnO]NPs was effectively mitigated by priming, which could be achieved through maintaining photosystem II activity and carbon fixation enzymes^65,66^. A key highlight of our findings is the high accumulation of soluble proteins and metabolites. This integration serves a dual function: osmotic adjustment and ionic sequestration. As typically folded soluble proteins are essential for functionality^67^, their induction upon priming suggests an enhanced capacity to chelate free Zn ions liberated from nanoparticles. This Metabolic Buffer is crucial for sustaining water potential and protecting biomolecules from oxidative denaturation under high-dose metal stress^67,68^.

The induction of enzymatic antioxidants (SOD, POD, CAT, APX) is a hallmark of the acquired resilience provided by SNP and LA. While high [ZnO]NPs levels typically deplete energy resources^69^, primed seedlings exhibited a robust Antioxidant Shield. Most importantly, the Metal Chelating Activity (reaching up to 98% in Table 1) represents the primary detoxification mechanism. By integrating the enzymatic defense with metal sequestration via phytochelatins and metallothioneins, the seedlings could be prevented the formation of highly toxic hydroxyl radicals through Fenton-type reactions^70–72^. This comprehensive machinery ensures that ROS levels remain below the threshold of irreversible oxidative damage.

From a practical perspective, the successive priming strategy (LA+SNP) offers a cost-effective and scalable tool for farmers in regions affected by industrial nano-pollution. This technique could potentially reduce the reliance on chemical soil remediators by fortifying the seed’s intrinsic defense systems before field emergence.

The computational data provides a molecular basis for understanding the observed resiliency of the wheat seedlings in the presence of [ZnO]NPs. ZnO’s high chemical reactivity is evident in its chemical hardness (η = 1.41 eV), which is very low, indicating that the ZnO surface is highly susceptible to modification by external chemical agents. The extremely negative adsorption energies for both LA and SNP imply that a stable complex is formed with these molecules, acting almost as a ‘molecular shield’ for the [ZnO]NPs. This shields the reactive surface area of the ZnO NPs, which from a physiological viewpoint effectively reduces the accessible reactive surface area. This reduces the [ZnO]NPs’ ability to interact with plant tissue and produce ROS.

The DFT data provides a molecular basis for understanding the increased metal chelating activity and reducing power observed for the [ZnO]NPs with LA and SNP. The nucleophilic nature of carboxyl and cyanide groups, evident in the MEP plots, enhances the ability of the wheat seedlings to chelate free metal ions, thereby enhancing the metal chelating activity—an essential step in heavy metal detoxification^73^.

The ‘molecular shielding’ also reduces the metabolic stress cost for the plant, thereby providing additional free energy for the accumulation of soluble metabolites such as proline and sugars, which play a crucial role in osmoregulation for the plant^74^. The marked enhancement in reducing power observed for the LA+SNP treated seedlings is consistent with the FMO analysis, where the high ability of these molecules to donate electrons is evident.

Thus, the higher distortion observed for the ZnO LA complex provides a molecular basis for understanding the enhanced activity observed for the [ZnO]NPs with LA in the priming experiments. Consequently, [ZnO]NPs -induced toxicity is averted through a dual action: the ZnO surface is made stable through strong adsorption with LA/SNP, a phenomenon consistent with the behavior observed for the modified ZnO system^75^; while the plant’s defenses are enhanced through a boost in chelating capacity and reducing power to scavenge the remaining oxidative stress.

### Study limitations and perspectives

Although the present study shows the effectiveness of successive LA and SNP priming in mitigating the [ZnO]NPs stress, some limitations need to be recognized. The first phase of investigation was based on the physiological, biochemical and quantum-chemical (DFT) aspects, while the molecular signaling networks at the transcriptomic level are still not fully elucidated. Second, the experiments were conducted under controlled laboratory conditions to decouple the effects of nanoparticle toxicity; however, the long-term stability and performance of these priming sequences in complex, multi-stress field environments needs further validation.

## Conclusion

The current study spotlights the transition of [ZnO]NPs dose from beneficial to acute phytotoxic concentration at higher doses. The findings suggest that the successive seed priming agents (LA+SNP) is a superior technique for counteracting [ZnO]NPs phytotoxicity in wheat seedlings when compared to individual priming agents. This synergistic mode of action of two redox-priming agents not only restores growth and photosynthetic machinery but also governed a robust detoxification shield by magnifying metal chelating activity and different ROS quenching abilities.

Frontier molecular orbital (HOMO–LUMO) analysis and molecular electrostatic potential (MEP) maps revealed favorable electronic complementarity between ZnO and the priming agents, indicating potential charge-transfer interactions at the adsorption interface. DFT calculations further confirmed the thermodynamically favorable adsorption of LA and SNP on the ZnO surface, evidenced by large negative adsorption energies and significant structural modifications of the Zn–O bond. These molecular interactions suggest effective surface passivation, which reduces the availability of reactive sites on the nanoparticles and contributes to the mitigation of their chemical reactivity.

This study outcomes strengthens the concept of the “Successive Priming” technique as an, effective, sustainable and feasible tool for securing food demands, particularly under soils contaminated circumstances with engineered nanomaterials. The result out comes emphasize the environmental and agricultural impact of adopting successive seed redox-priming technique as a high-precision strategy for nanometals detoxification and crop protection.

Further work should be directed towards elucidating the transcriptomic and proteomic signatures of successive redox-priming, to shed additional light on the genetic networks controlling nanoparticle detoxification. In addition, large-scale field trials are recommended to confirm the long-term efficacy of these priming sequences under various environmental conditions.

## Data Availability

All data generated or analyzed during this study are included in this published article and its supplementary information file is not publicly available due to its proprietary nature. Supporting data cannot be made openly available but are available from the corresponding author on reasonable request.

## Abbreviations

SNP: Sodium nitroprusside
NO: Nitric oxide
LA: α-Lipoic acid
[ZnO]NPs: Zinc oxide nanoparticles

## Declarations

### Generative AI and AI-assisted technologies in the writing process

During the preparation of this manuscript the author(s) used Gemini and Grammarly in order to improve the technical and English language of the paper. After using this tool/service, the author(s) reviewed and edited the content as needed and take(s) full responsibility for the content of the publication.
